# Letter to the Editor: “TP53 and KRAS co-mutations are associated with worse outcomes in mucinous ovarian carcinomas”

**DOI:** 10.3389/fonc.2025.1686095

**Published:** 2025-10-07

**Authors:** Lisa Feinstein

**Affiliations:** Camelot Animal Hospital, Davie, FL, United States

**Keywords:** ovarian mucinous carcinoma, ovarian mucinous adenocarcinoma (OMA), ovarian mucinous borderline tumor, mucinous adenoma surgery, cystectomie laparoscopique, mucinous cyst, mucinous ovarian cancer, mucinous cystadenocarcinoma

I am writing in response to “TP53 and KRAS co-mutations are associated with worse outcomes in mucinous ovarian carcinomas.” My daughter Kate was diagnosed at 18 years old with mucinous ovarian carcinoma (MOC) when the pathology from the laparoscopic cystectomy showed intraepithelial carcinoma amid the mostly mucinous adenoma. Before the cystectomy, the cyst showed no solid part on ultrasound, and because a previous (ipsilateral) cystectomy 18 months prior showed (benign) mucinous adenoma, the gynecologist assumed that it was another benign mucinous adenoma cyst and performed another laparoscopic cystectomy on the (18-cm) cyst. A gynecologic oncologist then performed an oophorectomy and staging surgery 8 weeks after the cystectomy, and mucinous carcinoma cells were found in the peritoneal washing and in a small portion of the infracolic omentum that was adhered to the cut surface of the ovary. Lymph nodes were negative. Pathology was read as expansile, well-differentiated mucinous ovarian carcinoma at stage 3, and the tumor genetics showed TP53 and KRAS G12V mutations. Kate underwent egg harvesting for storage and then completed six cycles of FOLFOX chemotherapy. Soon after, her CA125 climbed to 54 and a computed tomography (CT) scan showed omentum recurrence, which was confirmed with percutaneous biopsy, and she developed massive ascites and abdominal pain. Kate then had hyperthermic intraperitoneal chemotherapy (HIPEC) cytoreduction where mucinous cancer was found all over her abdomen (peritoneal carcinoma index of 39) including her entire intestinal tract. After surgery, she developed (high) bowel obstruction from tumor regrowth along the gastrointestinal tract. Because her tumor was Her2 positive, she was given Enhertu for three cycles, but it failed to reduce tumor burden in her abdomen and in her chest cavity, which was producing malignant pleural effusion. Kate died at 19 years old 1 year after staging. Expansile and borderline mucinous ovarian cancer are supposed to behave relatively benignly and not spread even with spillage. Yet, Zhang et al.’s findings showed that expansile primary mucinous ovarian carcinoma can behave aggressively, especially those with TP53 and KRAS mutations. There seems to be a disconnect between how mucinous cysts in young women are perceived by gynecologists and their potential pathogenicity. Gynecologists are not on the alert that a young woman in her teens or 20s may have a mucinous carcinoma or borderline cyst, and gynecologists may be upstaging these young women’s cancer by performing laparoscopic cystectomy. Preserving fertility and making keyhole incisions are factors that are weighing too heavily with gynecologists when deciding on the surgical approach of mucinous cysts and risk of malignancy. On the Mucinous Ovarian Cancer Facebook forum I subscribe to, there were many women who were fortunate enough to have a laparotomy oophorectomy or total abdominal hysterectomy with bilateral salpingo-oophorectomy (TAH-BSO) with no spillage even though their doctor did not suspect cancer, and their doctors were surprised when the pathology showed MOC. Likewise, there were many women like my daughter who had laparoscopic cystectomy on what was thought to be a benign mucinous cyst, and because of the spillage and cutting into the ovarian capsule, cancer spread into their abdominal cavity. My daughter’s mucinous cyst recurred on the same ovary 18 months after the first cystectomy, and the gynecologist performed another laparoscopic cystectomy. Other gynecologists I have spoken to said that they would have also performed a cystectomy the second time because she was 18 years old and pathology was benign at the first cystectomy. Yet, mucinous adenoma cyst recurrence is rare in the literature, and mucinous ovarian cysts follow a path towards malignancy, which most likely happened to my daughter. If Kate were assessed by a gynecological oncologist initially, the gynecological oncologist may have decided to perform a laparotomy oophorectomy and her mucinous tumor would have been removed intact without spillage. Scientific literature implies that mucinous ovarian cancer is a slow-growing cancer, and that expansile pattern suggests that it will not likely spread. Yet, we experienced the total opposite, with my daughter Kate’s cancer acting very aggressively and spreading all over her abdomen, most likely because her tumor was not surgically contained and it carried TP53 and KRAS mutations. Gynecologists need to become more aware of the dangers of large mucinous cysts in women and refer to a gynecologic oncologist for their opinion before performing laparoscopic cystectomy that can potentially spread and upstage mucinous cancer.

